# Transient post-operative overexpression of CXCR2 on monocytes of traumatic brain injury patients drives monocyte chemotaxis toward cerebrospinal fluid and enhances monocyte-mediated immunogenic cell death of neurons in vitro

**DOI:** 10.1186/s12974-022-02535-6

**Published:** 2022-06-29

**Authors:** Huayang Wang, Qibing Huang, Zhijie Zhang, Jian Ji, Tao Sun, Donghai Wang

**Affiliations:** 1 Department of Clinical Laboratory, Qilu Hospital of Shandong University, Jinan, China; 2 Department of Neurosurgical Intensive Care Unit, Qilu Hospital of Shandong University, Jinan, China; 3 Department of Ultrasound, Shandong Maternal and Child Health Hospital, Jinan, China; 4 Department of Neurosurgery, Qilu Hospital of Shandong University, # 107 Wenhuaxi Road, Jinan, Shandong China

**Keywords:** Traumatic brain injury, Monocytes, CXCR2, Chemotaxis, Immunogenic cell death

## Abstract

**Background:**

After traumatic brain injury (TBI), peripheral monocytes infiltrate into the central nervous system due to disruption of the blood–brain barrier, and play an important role in neuroinflammation. However, the mechanisms regulating the movement and function of peripheral monocytes after TBI have not been fully investigated.

**Methods:**

TBI patients who underwent surgery at our hospital were recruited. CXCR2 expression in CD14^+^ monocytes from peripheral blood and cerebrospinal fluid (CSF) of TBI patients around surgery was analyzed by flow cytometry and compared with that of patients who suffered TBI 2–24 months prior and underwent cranioplasty. In vitro, serum or CSF from TBI/non-TBI patients were used to treat peripheral monocytes isolated from healthy volunteers to evaluate their effect on CXCR2 expression. Transwell experiments were performed to analyze the role of CXCR2 in monocyte chemotaxis toward the CSF. The role of CXCR2 in monocyte-mediated immunogenic cell death (ICD) of nerve cells was explored in an indirect co-culture system.

**Results:**

Transient CXCR2 upregulation in monocytes from the peripheral blood and CSF of TBI patients was detected soon after surgery and was associated with unfavorable outcomes. TBI serum and CSF promoted CXCR2 expression in monocytes, and dexamethasone reversed this effect. Peripheral monocytes from TBI patients showed enhanced chemotaxis toward the CSF and increased inflammatory cytokine secretion. The CXCR2 antagonist SB225002 decreased monocyte chemotaxis toward TBI CSF, and lowered pro-inflammatory cytokine secretion in monocytes treated with TBI serum. SB225002 also relieved ICD in nerve cells co-cultured with TBI serum-treated monocytes.

**Conclusions:**

CXCR2 is transiently overexpressed in the peripheral monocytes of TBI patients post-surgery, and drives peripheral monocyte chemotaxis toward CSF and monocyte-mediated ICD of nerve cells. Therefore, CXCR2 may be a target for monocyte-based therapies for TBI.

**Supplementary Information:**

The online version contains supplementary material available at 10.1186/s12974-022-02535-6.

## Introduction

Traumatic brain injury (TBI) is a significant health concern affecting over 50 million patients annually and is particularly prevalent in young adults [1]. It is the leading cause of death and persistent neurocognitive impairment. The outcome of TBI is primarily determined by two phases of pathophysiological mechanisms: primary and secondary injury. Primary injury refers to the mechanical injury of brain tissue caused by external violence immediately after injury; secondary injury refers to nerve cell dysfunction, apoptosis, and necrosis caused by inflammation, oxidative stress, disruption of the blood–brain barrier (BBB), and compression of hematoma/edema [2]. The primary injury is considered irreversible; however, secondary injury can be interfered and is therefore attractive for improving outcomes.

Inflammatory factors and chemokines are released immediately after the occurrence of moderate TBI and peak within a few hours of injury [3]. Neuroinflammation causes degeneration and cell death. Inflammatory substances released by dead nerve cells activate pattern recognition receptors and further stimulate the inflammatory response [4]. Therefore, cell death and inflammation are the core mechanisms of secondary injury in TBI. Immunogenic cell death (ICD), including pyroptosis, ferroptosis, and necroptosis, is a recently discovered programmed necrotic cell death characterized by the disruption of mitochondrial permeability [5]. Interventions targeting various biological mechanisms of ICD, from upstream signaling pathways to downstream inflammatory cytokines or inflammasomes, have been developed for TBI treatment [5]. These interventions effectively inhibit ICD in nerve cells; however, no therapy has been proven to improve the prognosis of TBI in clinical trials.

Due to the disruption and dysfunction of the BBB, peripheral monocytes infiltrate into the central nervous system (CNS) after TBI and play an important role in neuroinflammation [6]. Therefore, another potential therapeutic strategy for TBI is to inhibit monocyte recruitment by interrupting the interaction between chemokine receptors and their corresponding ligands. CCR2 is the most well-studied chemokine receptor in this field, as it is required for monocytes to enter the CNS. Different studies have shown that targeting CCR2 or its ligands can inhibit monocyte chemotaxis and improve short- and long-term outcomes [7, 8]. However, another study has shown that CCR2 deletion in a TBI mouse model caused abnormally increased tau protein phosphorylation in the cortex and hippocampus, which is closely related to the loss of physiological function and the acquisition of pathological function in various neurodegenerative diseases [9]. This suggests that monocyte subpopulations may differentially influence the outcome, which remains largely unknown.

CXCR2 is the main chemokine receptor of neutrophils and interacts with various chemokines, including CXCL1-3, and CXCL5-8. CXCR2 plays an important role in the inflammatory response by mediating neutrophil recruitment. In the CNS, CXCR2 is mainly expressed in neurons [10]. Reduced nerve cell apoptosis with improved neurological behaviors was observed in a TBI mouse model with the application of a CXCR2 antagonist [11]. However, there has been little research on CXCR2 expression in the monocytic system. In the present study, we analyzed dynamic changes in CXCR2 expression in monocytes in the peripheral blood and cerebrospinal fluid (CSF) of TBI patients and found a transient CXCR2 upregulation in monocytes following TBI, which was associated with poorer short-term outcomes. In vitro, TBI serum and CSF promoted CXCR2 expression in monocytes, and dexamethasone reversed this effect. CXCR2 drove peripheral monocyte recruitment toward TBI CSF, drove a pro-inflammatory phenotype in TBI serum-treated monocytes, and enhanced monocyte-mediated ICD of nerve cells, initiated by oxidative stress. Our results suggest that CXCR2 may be a target for monocyte-based therapies for TBI.

## Materials and methods

### Patients and sample collection

This study was conducted in accordance with the Declaration of Helsinki and was approved by the Human Research Ethics Committee of Qilu Hospital of Shandong University (China). All patients provided consent for sample collection and subsequent analyses. The inclusion and exclusion criteria of patients and healthy controls involved in this study were as follows: (1) TBI group, patients who were presented to our hospital between July, 2018 and November, 2020, with Glasgow Coma Score (GCS) 3–15 on admission, and underwent hematoma aspiration, craniotomy, or decompressive craniectomy in our hospital. Patients who had any previous disabling neurological disease, previous craniectomy, spinal cord injury, penetrating head injury were excluded. Patient demographic and clinical data are recorded as Additional file 1: Table S1. (2) Crainoplasty group, patients who had cranial defects due to decompressive craniectomy against TBI 2–24 months ago, and underwent crainoplasty in our hospital. Patients had GCS 8–15 on admission, with no previous crainoplasty history and no sign of hemodynamic or respiratory instability, intracranial or systemic infection, or delayed wound healing from the initial surgeries or other trauma-related surgeries. After crainoplasty, the patients showed no sign of intracranial or systemic infection in hospital. (3) Non-TBI group, patients who had seizure history and were diagnosed as idiopathic epilepsy in our hospital. Brain CT and/or MRI showed no evidence of any lesions on brain, and no other causes for the seizures could be identified. (4) Healthy control group, people who underwent physical examinations in our hospital and denied any chronic disease history. No obvious abnormalities were identified in the physical examination results. The clinical data of the patients were recorded. Blood and CSF samples remaining after laboratory testing were collected.

### Flow cytometry analysis

For phenotyping human circulating monocytes, peripheral blood mononuclear cells (PBMCs), which contain lymphocytes and monocytes, but no neutrophils, were obtained from the anticoagulated peripheral blood of TBI patients or patients who underwent cranioplasty by centrifugation with Ficoll-Paque Plus (Sigma-Aldrich, MO, USA). PBMCs were stained with anti-human CD16 (Clone: 3G8)-FITC (BioLegend, CA, USA, Cat: 302006), CD14 (Clone: MΦP9)-PE (Becton Dickinson, NJ, USA, Cat: 347497), and CXCR2 (Clone: 5E8/CXCR2)-APC (BioLegend, Cat: 320710) fluorescent monoclonal antibodies (mAb). For phenotyping monocytes in the CSF, 10 mL of anticoagulated CSF was obtained from TBI or non-TBI patients. Nucleated cells were collected by centrifugation at 150 g for 10 min, and then stained with anti-human CD11b (Clone: ICRF44)-FITC (BioLegend, Cat: 301330), CD14 (Clone: MΦP9)-PE (Becton Dickinson, Cat: 347497), and CXCR2 (Clone: 5E8/CXCR2)-APC (BioLegend, Cat: 320710) fluorescent mAb. To analyze surface calreticulin (CRT) expression, SH-SY5Y cells were stained with anti-human CRT (Clone: 1G6A7)-APC (Novus Biologicals, CO, USA, Cat: NBP1-47518APC). The isotype controls were analyzed in parallel. The samples were acquired on a FACSCalibur flow cytometer (BD Biosciences, NJ, USA) and analyzed using FlowJo software (FlowJo, OR, USA).

### Isolation and culture of human peripheral blood monocytes

CD14^+^ monocytes were isolated from PBMCs of healthy volunteers, TBI patients, or non-TBI patients by positive selection using anti-CD14-conjugated magnetic microbeads (Miltenyi Biotech, North Rhine-Westphalia, Germany) according to the manufacturer’s instructions. The purity was determined using flow cytometry, and was > 95%. The isolated primary peripheral monocytes were cultured in RPMI 1640 medium supplemented with 10% FBS, 100 U/mL penicillin, and 100 mg/mL streptomycin for subsequent experiments. To analyze the effect of TBI/non-TBI serum/CSF on phenotypes or cytokine secretion by monocytes, 1 × 10^6^ peripheral monocytes from healthy volunteers were treated with RPMI 1640 medium supplemented with TBI/non-TBI serum/CSF (20%, v:v) for 24 h. In certain experiments, SB225002 (10 μM, Cat: S7651, Selleck Chemicals, TX, USA) and/or lipopolysaccharide (LPS; 100 ng/mL, L3012, Sigma-Aldrich) were added to the medium. The neuroblastoma cell line SH-SY5Y was cultured in DMEM/F12 medium supplemented with 10% FBS, 100 U/mL penicillin, and 100 mg/mL streptomycin at 37 °C in an incubator with 5% CO_2_.

### Quantitative RT-PCR

RNA from human circulating CD14^+^ monocytes was extracted using the TRIzol Reagent (Invitrogen, MA, USA). cDNA was synthesized by reverse transcription. Quantitative RT-PCR (RT-qPCR) was conducted on a LightCycler 2.0 Instrument (Roche, Basel, Switzerland). GAPDH was used as an internal control. The primer sequences were as follows: cxcr2, forward: 5′-CAG TTA CAG CTC TAC CCT GCC-3′, reverse: 5′-CCA GGA GCA AGG ACA GAC CCC-3′; nf-κb p65, forward: 5′- CTG CAG TTT GAT GAT GAA GA-3′, reverse: 5′- TAG GCG AGT TAT AGC CTC AG-3′; il-1b, forward: 5′- TGA TGG CTT ATT ACA GTG GCA ATG -3′, reverse: 5′- GTA GTG GTG GTC GGA GAT TCG -3′; il-6, forward: 5′-CAC ACA GAC AGC CAC TCA CC-3′, reverse: 5′-GCT CTG GCT TGT TCC TCA CT-3′; il-8, forward: 5′-TCT GCA GCT CTG TGT GAA GG-3′, reverse: 5′-TGA ATT CTC AGC CCT CTT CAA-3′; il-10, forward: 5′-CAC TGC TCT GTT GCC TGG T-3′, reverse: 5′-TCT GGG TCT TGG TTT TCA GC-3′; il-12p40, forward: 5′-GGA AGC ACG GCA GCA GAA TA-3′, reverse: 5′-AAC TTG AGG GAG AAG TAG GAA TGG-3′; ifn-g, forward: 5′- TGT AGC GGA TAA TGG AAC TCT TTT-3′, reverse: 5′- AAT TTG GCT CTG CAT TAT T-3′; tnf-a, forward: 5′-ATG AGC ACT GAA AGC ATG ATC C-3′, reverse: 5′-GAG GGC TGA TTA GAG AGA GGT C-3′; gapdh, forward: 5′-TCG GAG TCA ACG GAT TTG GTC GTA-3′, reverse: 5′-CTT CCT GAG TAC TGG TGT CAG GTA-3′.

### Chemotaxis assay

Peripheral CD14^+^ monocytes (1 × 10^5^) from TBI or non-TBI patients were added to the upper compartment of the Transwell inserts (24-well plate, 5 μm pores; Corning, NY, USA) in 100 μL of serum-free medium. RPMI 1640 medium supplemented with CSF of TBI/non-TBI patients (50%, v: v) or SH-SY5Y conditioned medium (600 µL) was added to the lower chamber of the plate. In certain conditions, peripheral monocytes were pretreated with SB225002 for 2 h before they were added to the upper compartment. After incubation at 37 °C for 16 h, the migrated cells on the lower surface of the filter were fixed in 10% formalin and stained with crystal violet. Five random fields of each well were photographed, and the cells were counted.

### Enzymes linked immunosorbent assay (ELISA)

Human IL-1β (Cat: H0149c, Elabscience Biotech, Wuhan, China), IL-10 (Cat: E-EL-H0103c, Elabscience Biotech), TNF-α (Cat: E-EL-H0109c, Elabscience Biotech), CXCL1 (Cat: CSB-E09150h, Cusabio, Wuhan, China), CXCL2 (Cat: CSB-E07420h, Cusabio), CXCL8 (Cat: H6008, Elabscience Biotech), and HMGB-1 (Cat: E-EL-H1554, Elabscience Biotech) levels in CSF or cell culture supernatants were quantified using a commercial ELISA kit according to the manufacturer’s instructions.

### Apoptosis assay

To test the role of CXCR2 in monocyte-mediated nerve cell apoptosis, SH-SY5Y cells (1 × 10^5^) were seeded in a six-well plate and treated with H_2_O_2_ (50 μM) for 5 min, and then the medium was refreshed. A 0.4 μm pore size Transwell chamber was inserted into the well, and peripheral monocytes (1 × 10^6^) treated with TBI/non-TBI serum, were seeded into the upper chamber to establish an indirect co-culture system. After 24 h, SH-SY5Y cells were stained with FITC Annexin V Apoptosis Detection Kit I (Cat: 556,547, BD Pharmingen, CA, USA) according to the manufacturer’s instructions. In some experiments, SB225002 (10 μM) was also added into the co-culture system along with peripheral monocytes.

### Immunofluorescence staining

For immunofluorescent staining of HMGB1, SH-SY5Y cells co-cultured with monocytes were fixed in cold acetone/methanol (1:1) and permeabilized with 0.1% Triton X-100 in PBS. The fixed cells were incubated with HMGB1 rabbit antibody (Cat: 3935, Cell Signaling Technology, MA, USA) followed by incubation with goat anti-rabbit IgG H&L (Alexa Fluor® 488, Cat: ab150077, Abcam, Cambridge, UK). Nuclear staining was conducted with 10 μg/mL DAPI (C0065, Solarbio, Beijing, China). The fluorescence was monitored using an inverted fluorescence microscope (IX81, Olympus, Tokyo, Japan) with a microscope digital camera (DP73, Olympus). Images were adjusted using Adobe Photoshop software.

### ATP assay

Extracellular ATP concentration in SH-SY5Y supernatant was analyzed using an ATP Chemiluminescence Assay Kit (Cat: E-BC-F002, Elabscience) according to the manufacturer’s instructions.

### Statistical analysis

Student’s t-test and analysis of variance were used for statistical comparisons. The linear correlation between two variables was determined using Pearson’s correlation. Differences in variables between TBI patients with favorable/unfavorable short-term outcomes were determined using the cross-tabulation analysis with the χ2-test or Fisher’s exact test. The specific statistical test for each experiment is shown in the figure captions. All statistical analyses were conducted using the SPSS software version 13.0. Statistical significance was set at *P* < 0.05.

## Results

### The perioperative expression profile of CXCR2 on circulating monocytes of TBI patients

**Fig. 1:**
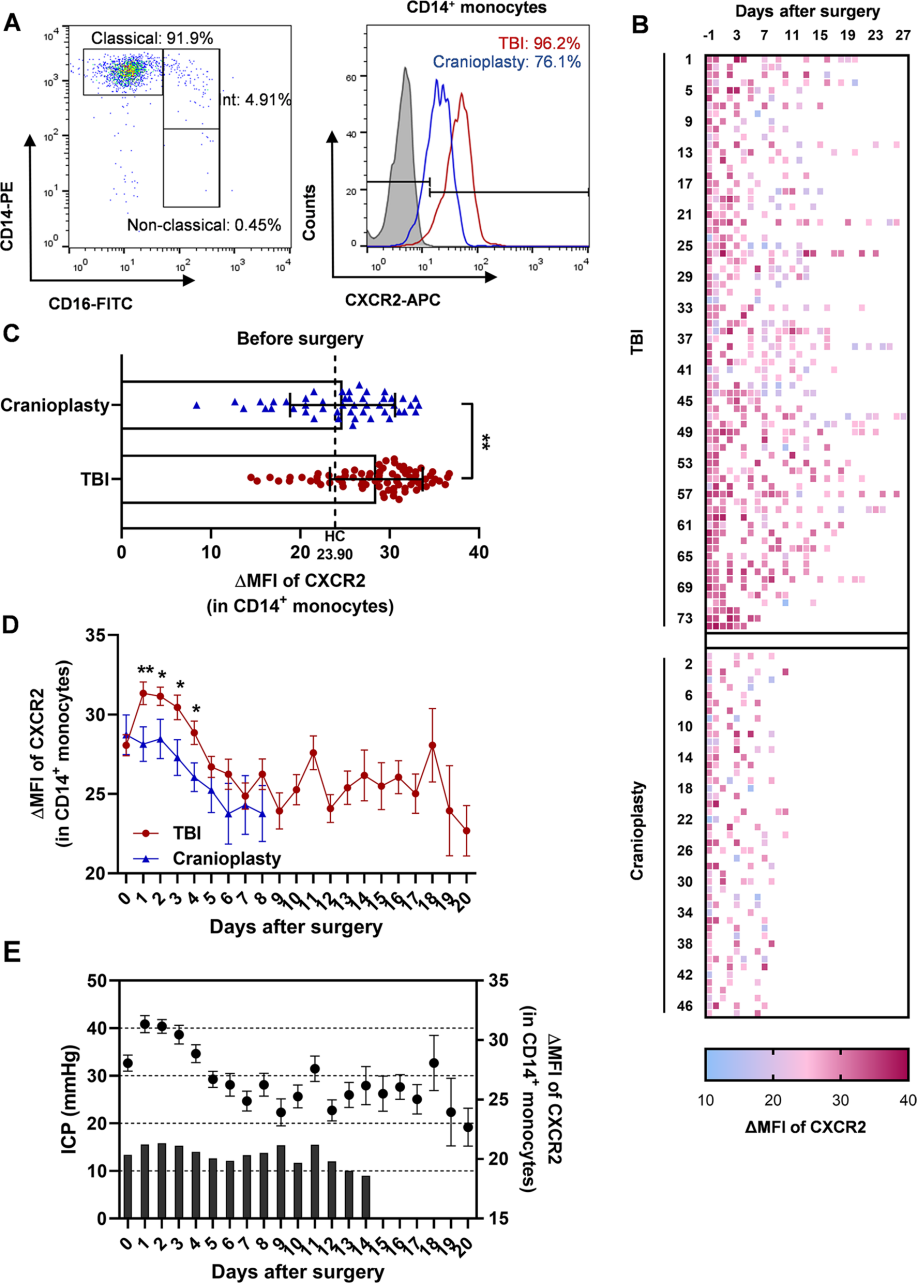
CXCR2 expression in peripheral monocytes is transiently upregulated in traumatic brain injury (TBI) patients after surgery. **A** (Left) Flow cytometry results of constitution of classical (CD14^++^CD16^−^), intermediate (CD14^++^CD16 +), and non-classical (CD14^low^CD16^+^) monocyte subsets in peripheral blood of TBI patients after surgery. (Right) Flow cytometry results of CXCR2 expression on peripheral classical monocytes of patients with TBI or crainoplasty. Isotype control is shown as gray filled histogram and CXCR2 is shown as open histogram. **B** The expression profile of ΔMFI value of CXCR2 in peripheral CD14^+^ monocytes around surgery of patients with TBI or crainoplasty. **C** ΔMFI value of CXCR2 in peripheral CD14^+^ monocytes of patients with TBI or crainoplasty before surgery. The dashed line represents average ΔMFI value of CXCR2 in monocytes of healthy controls (HC, *n* = 29). **P < 0.01, determined by Mann–Whitney tests. **D** ΔMFI value of CXCR2 on peripheral CD14^+^ monocytes of patients with TBI or crainoplasty after surgery. *P < 0.05, **P < 0.01, determined by Mann–Whitney tests. **E** CXCR2 ΔMFI value and intracranial pressure (ICP) of patients with TBI after surgery. The dot plot represents the CXCR2 ΔMFI value and the column represents ICP. Data are presented as mean with SEM

To explore the association of monocyte-expressed CXCR2 with the pathophysiology of TBI, we first detected the CXCR2 expression level on monocytes in the peripheral blood of TBI patients during the perioperative period. Patients who underwent cranioplasty were included in the control group. According to the expression levels of CD14 and CD16, circulating monocytes can be divided into three subsets: classical (CD14^hi^CD16^−^), intermediate (CD14^hi^CD16^+^), and non-classical (CD14^lo^CD16^+^). The classical subset accounted for > 90% of circulating monocytes in TBI patients, and the proportion of non-classical subsets was small (Fig. 1A). Therefore, in subsequent experiments, we analyzed the expression levels of CXCR2 on CD14^+^ circulating monocytes, which contain classical and intermediate subsets. We analyzed the perioperative mean fluorescence intensity (MFI) values of CXCR2 on monocytes in 74 patients with TBI and 47 patients who underwent cranioplasty (Fig. 1B). Before surgery, the MFI of CXCR2 in the monocytes of TBI patients was significantly higher than that of patients who underwent cranioplasty, and the latter was close to that of healthy controls (represented as dashed line) (Fig. 1C). On the surgery day (day 0), there was no significant difference in the MFI of CXCR2 between patients with TBI and cranioplasty. However, on day 1 after surgery, the MFI of CXCR2 in monocytes of TBI patients increased significantly. In contrast, CXCR2 MFI values did not increase in patients who underwent cranioplasty after surgery. Monocytes from TBI patients showed higher MFI values of CXCR2 from day 1 to day 4 after surgery than those from patients who underwent cranioplasty. On day 7 after surgery, the MFI of CXCR2 in monocytes of the two groups tended to be equivalent (Fig. 1D). We further analyzed the relationship between intracranial pressure (ICP) and CXCR2 expression in TBI monocytes. The results showed that the average ICP of TBI patients fluctuated within a certain range, decreased gradually after being at a relatively high level 1–3 days after surgery, and reached a relatively low level on day 6 after surgery. This trend was consistent with the trend of CXCR2 expression intensity (Fig. 1E).

To further explore the effect of TBI or surgery on the expression of CXCR2 in circulating monocytes, TBI patients were divided into three groups as ≤ 12 h, 13-24 h and > 24 h according to the intervals from TBI to surgery. There was no significant difference in the pre-operative MFI value of CXCR2 between three groups (Additional file 2: Fig. S1A). However, in the analysis of post-operative CXCR2 expression, we observed that the upregulation of CXCR2 in > 24 h group appeared later after surgery and decreased rapidly (Additional file 2: Fig. S1B). To further confirm our observations, we compared the average values of CXCR2-positive percentage and MFI within 3 days and 4–5 days after surgery between the three groups. The results showed that CXCR2 expression in 13-24 h group was higher than that in > 24 h group within 3 days after surgery. Besides that, CXCR2 expression in 13-24 h group was also slightly higher than that in ≤ 12 h group within 3 days after surgery, but significant difference was not reached (Additional file 2: Fig. S1C). Next, we divided TBI patients into three groups as aspiration, craniotomy and decompressive craniectomy according to different operation styles. No significant differences were found in pre- or post-operative CXCR2 expression in the three groups (Additional file 3: Fig. S2).

**Fig. 2:**
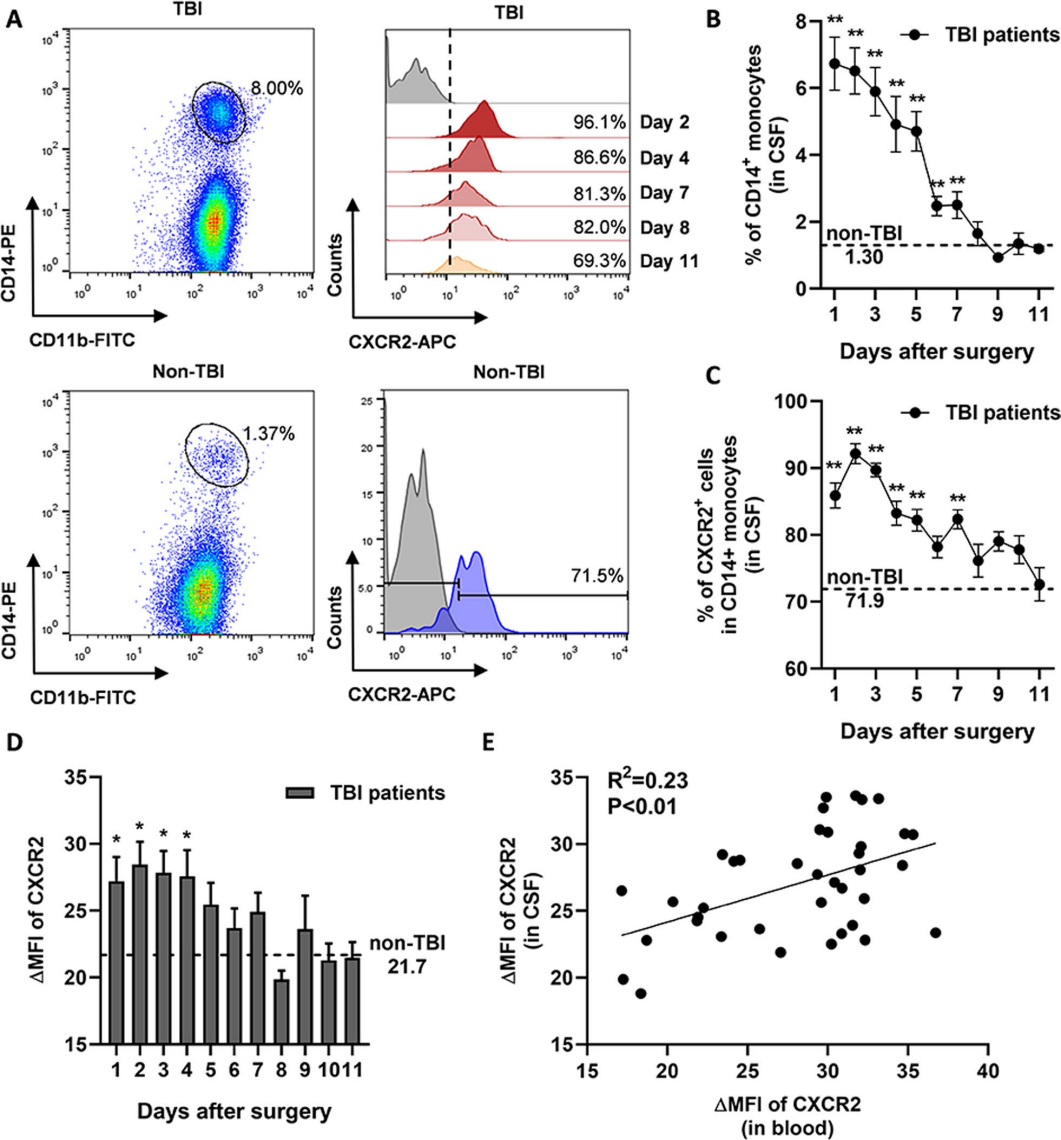
CXCR2 expression is upregulated in monocytes in cerebrospinal fluid (CSF) of TBI patients post-operation, and correlated with the CXCR2 expression value on peripheral monocytes.** A** (Left) Flow cytometry results of CD11b^+^CD14^+^ monocyte subsets in CSF of TBI patients after surgery. (Right) Typical flow cytometry results of CXCR2 expression on CD14^+^ monocyte subsets in CSF of TBI patients on different days after surgery. **B** Proportion of CD14^+^ monocytes in CSF of TBI or non-TBI patients. The dashed line represents average proportion of CD14^+^ monocytes of non-TBI patients. **P < 0.01, TBI vs. non-TBI, determined by unpaired *t* test. **C** Proportion of CXCR2^+^ subsets in CD14^+^ monocytes in CSF of TBI or non-TBI patients. The dashed line represents average proportion of CXCR2^+^ subsets of non-TBI patients. **P < 0.01, TBI vs. non-TBI, determined by unpaired *t* test. **D** ΔMFI value of CXCR2 on CD14^+^ monocytes in CSF of TBI or non-TBI patients. The dashed line represents average ΔMFI value of CXCR2 of non-TBI patients. *P < 0.05, TBI vs. non-TBI, determined by unpaired *t* test. **E** Linear association of ΔMFI value of CXCR2 between monocytes in CSF and monocytes in peripheral blood of TBI patients. *R*^2^ was determined by Pearson correlation. Data are presented as mean with SEM

After TBI occurs, monocytes in the peripheral circulation enter the CNS due to destruction of the BBB. Therefore, we detected CD14^+^ monocytes in the CSF of TBI patients and the expression of CXCR2, and compared with those in the CSF of non-TBI (idiopathic epilepsy) patients (Fig. 2A). As shown in Fig. 2B, the proportion of CD14^+^ monocytes in CSF of TBI patients decreased after surgery, from 6.73% on day 1 to 2.50% on day 7 after surgery. After 7 days, the proportion of CD14^+^ monocytes in CSF of TBI patients was similar to that of non-TBI patients. The proportion of CXCR2^+^ subsets in CD14^+^ monocytes in the CSF of TBI patients is approximately 85.9% one day after surgery, increased to 92.1% two days after surgery, and then decreased and stabilized approximately 8 days after surgery, which was close to the proportion of CXCR2^+^ subsets in CSF of non-TBI patients (Fig. 2C). The MFI value of CXCR2 was consistent with its positive proportion (Fig. 2D). Correlation analysis was performed on the MFI of CXCR2 between circulating monocytes and monocytes in the CSF of the same TBI patient, and a positive correlation was found (Fig. 2E).

### The post-operative CXCR2 expression on monocytes is different in TBI patients with different short-term prognosis

The above results suggest that CXCR2 expression on monocytes is upregulated in TBI patients, therefore, we further explored whether the expression level of CXCR2 is related to the prognosis of TBI. We used the Glasgow Outcome Score (GOS) at discharge to evaluate the short-term patient prognosis. Patients with a score of 1–3 were regarded as having an unfavorable prognosis and a score of 4–5 were regarded as having a favorable prognosis. As shown in Table 1, the average age of patients with a favorable prognosis is lower than that of patients with an unfavorable prognosis. Dilated pupil diameter, sluggish pupil activity, lower Glasgow Coma Score (GCS) on admission, and tracheotomy during hospitalization were all related to unfavorable short-term prognosis.

**Table 1:** Relationship of categorical variables to discharge outcome postoperatively

Variables	Discharge GOS score		*p* value
	Unfavorable	Favorable	
	(*n* = 30)	(*n* = 44)	
Gender			
Male	25	36	0.87
Female	5	8	
Age (years)			
	55.60 ± 14.55	47.68 ± 15.34	0.03
Hospital day (days)			
	18.30 ± 8.84	17.57 ± 6.70	0.76
Injury mechanism			
Fall	13	25	0.18
Traffic accident	12	17	
Others	5	2	
Dilated pupil			
Diameter of both pupils < 4 mm	17	41	< 0.01
Diameter of 1 pupil ≥ 4 mm	5	1	
Diameter of both pupils ≥ 4 mm	8	2	
Pupillary reactivity			
Brisk	7	27	< 0.01
Sluggish	9	13	
Nonreactive	14	4	
GCS score (admission)			
3–8	25	17	< 0.01
9–12	3	13	
13–15	2	14	
Injury style			
Unilateral	22	36	0.38
Bilateral	8	8	
Interval from injury to surgery (h)			
≤ 12	8	11	0.65
13–24	13	14	
25–48	2	5	
> 48	7	13	
Operation style			
Hematoma aspiration	10	17	0.12
Craniotomy	6	16	
Decompressive craniectomy	14	11	
Tracheotomy			
Yes	14	4	< 0.01
No	16	40	
WBC# (before surgery)			
	12.76 ± 5.10	15.01 ± 5.94	0.09
WBC# (after, first 3 days)			
	8.47 ± 3.36	9.61 ± 3.49	0.02
NEU# (before surgery)			
	11.06 ± 4.97	13.06 ± 5.36	0.11
NEU# (after, first 3 days)			
	7.07 ± 2.94	8.02 ± 3.24	0.03
LYM# (before surgery)			
	1.09 ± 1.06	1.19 ± 1.07	0.45
LYM# (after, first 3 days)			
	0.68 ± 0.34	0.82 ± 0.42	0.03
NLR (before surgery)			
	16.41 ± 12.81	15.40 ± 10.61	0.73
NLR (after, first 3 days)			
	12.66 ± 8.31	13.05 ± 9.45	0.68
MON# (before surgery)			
	0.56 ± 0.29	0.79 ± 0.40	< 0.01
MON# (after, first 3 days)			
	0.50 ± 0.30	0.66 ± 0.37	< 0.01
ALB (g/L, before, surgery)			
	39.41 ± 5.90^a^	39.47 ± 6.22^b^	0.82
CD14^+^ CXCR2^+^ monocytes (%)			
(before surgery)	88.98 ± 4.25	87.88 ± 4.82	0.29
ΔMFI of CXCR2 in monocytes			
(before surgery)	29.08 ± 5.26	28.10 ± 5.16	0.33
CD14^+^ CXCR2^+^ monocytes (%)			
(after, first 3 days)	91.45 ± 2.67	85.53 ± 4.65	< 0.01
ΔMFI of CXCR2 in monocytes			
(after, first 3 days)	32.62 ± 2.78	27.27 ± 4.23	< 0.01
CD14^+^ CXCR2^+^ monocytes (%)			
(after, 4–5 days)	89.42 ± 4.60	87.46 ± 4.00	< 0.01
ΔMFI of CXCR2 in monocytes			
(after, 4–5 days)	30.66 ± 4.54	27.49 ± 3.52	< 0.01

^a^*n* = 29; ^b^n = 42

There was no significant difference in CXCR2-positive percentage and MFI value of peripheral monocytes in patients with different prognoses at admission. However, in patients with a favorable prognosis, the mean CXCR2-positive percentage and MFI on monocytes during the first 3 days after surgery were lower than those with an unfavorable prognosis. The mean positive percentage and MFI of CXCR2 4–5 days after surgery were also lower in patients with a favorable prognosis (Table 1).

### Serum and CSF of TBI patients promote CXCR2 expression on monocytes through the NF-κB pathway

**Fig. 3:**
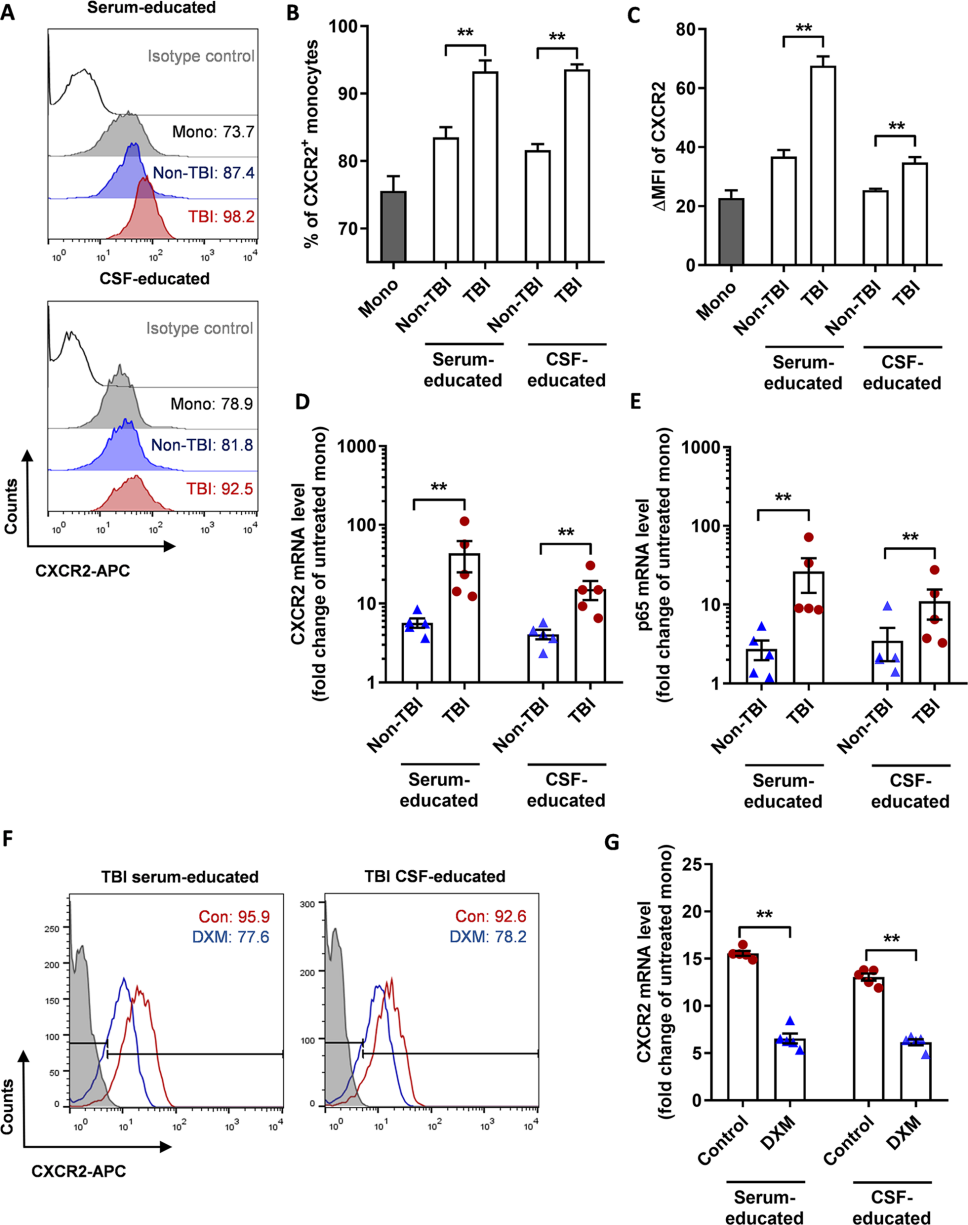
TBI serum and CSF promotes CXCR2 expression in peripheral monocytes. **A** Typical flow cytometry results of CXCR2 surface expression on peripheral monocytes educated by TBI/non-TBI serum (left) or CSF (right). Mono represents peripheral monocytes alone, not being educated by serum or CSF. **B** Percentage of CXCR2^+^ cells in peripheral monocytes alone, or educated by TBI/non-TBI serum or CSF. **P < 0.01, determined by paired *t* tests. **C** ΔMFI value of CXCR2 in peripheral monocytes alone, or educated by TBI/non-TBI serum or CSF. **P < 0.01, determined by paired *t* tests. **D** mRNA levels of CXCR2 in peripheral monocytes educated by TBI/non-TBI serum or CSF. Data are presented as fold change of CXCR2 mRNA expression in treated ones to that in untreated ones. **P < 0.01, determined by paired *t* tests. **E** mRNA levels of p65 in peripheral monocytes educated by TBI/non-TBI serum or CSF. Data are presented as fold change of p65 mRNA expression in treated ones to that in untreated ones. **P < 0.01, determined by paired *t* tests. **F** Typical flow cytometry results of CXCR2 surface expression on peripheral monocytes educated by TBI serum (left) or CSF (right), with/without dexamethasone (DXM) treatment. **G** mRNA levels of CXCR2 in peripheral monocytes educated by TBI serum or CSF, with/without DXM treatment. Data are presented as fold change of CXCR2 mRNA expression in serum/CSF-treated ones to that in untreated ones. **P < 0.01, determined by paired t tests. Data are presented as mean with SEM

We further explored the possible mechanism of CXCR2 upregulation in the monocytes of TBI patients. For this purpose, we isolated CD14^+^ monocytes from the peripheral blood of healthy volunteers and cultured them in the serum or CSF of TBI and non-TBI patients, respectively. These cells were hereinafter referred to as TBI/non-TBI-educated monocytes. The serum or CSF of TBI patients promoted a positive percentage (Fig. 3A, B) and expression intensity of CXCR2 (Fig. 3C) in monocytes compared with those of non-TBI patients. At the transcriptional level, the CXCR2 mRNA level in monocytes cultured in the serum/CSF of TBI patients was significantly higher than that of non-TBI patients (Fig. 3D).

The NF-κB pathway promotes CXCR2 expression. Therefore, we analyzed p65 mRNA levels in monocytes after culturing in serum/CSF of TBI/non-TBI patients. As shown in Fig. 3E, compared with that of non-TBI patients, the p65 mRNA level is significantly upregulated in monocytes after culturing in the serum/CSF of TBI patients. Dexamethasone is well recognized as an NF-κB pathway inhibitor. However, its role in TBI treatment remains controversial. We pretreated monocytes with dexamethasone before supplementing with TBI serum/CSF, and found that dexamethasone effectively reversed the promoting effect of TBI serum/CSF on CXCR2 expression in monocytes, presented both as a positive percentage and ΔMFI (Fig. 3F, G).

### CXCR2 drives monocyte chemotaxis toward the CSF of TBI patients

**Fig. 4:**
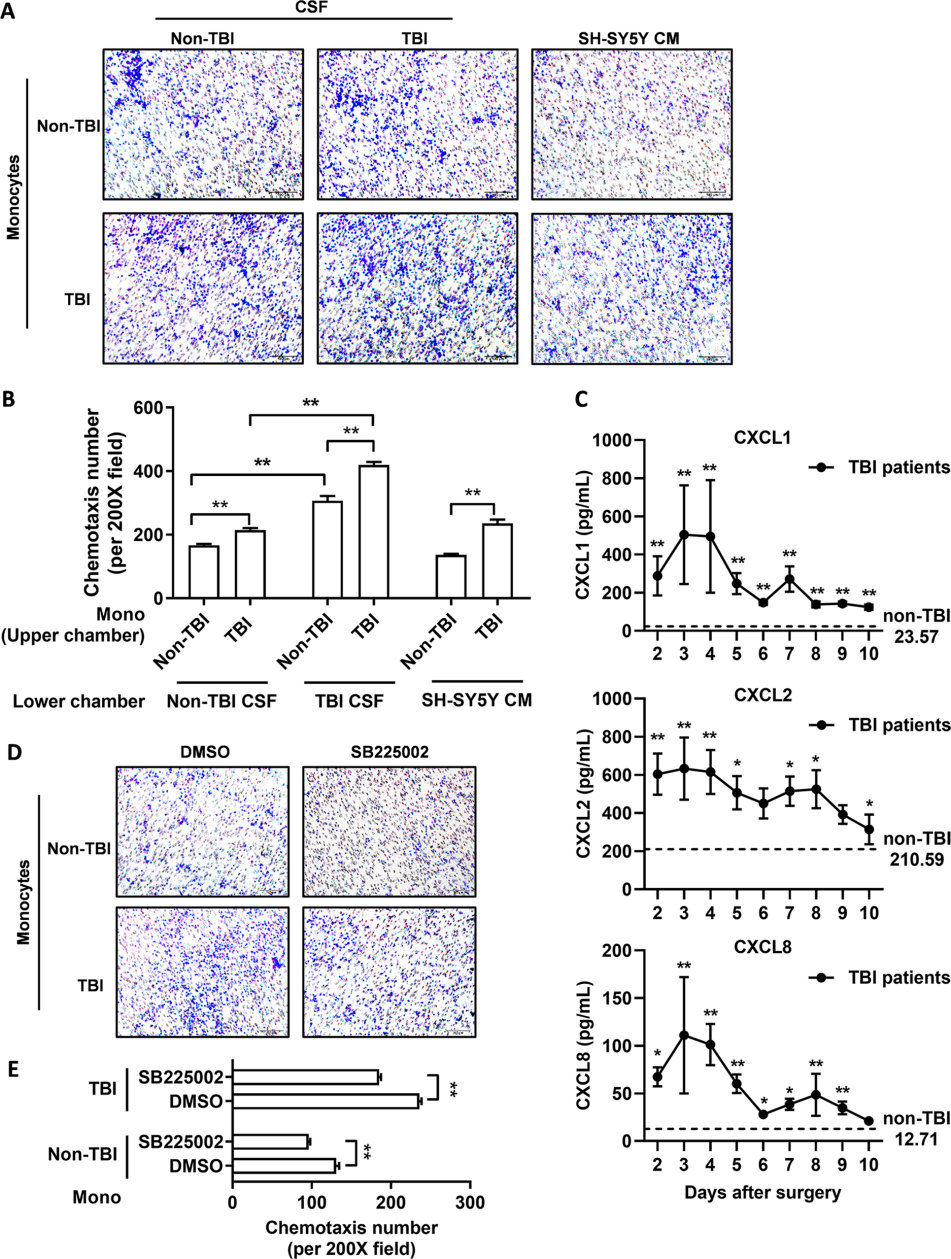
CXCR2 drives the chemotaxis of peripheral monocytes toward TBI CSF. **A** Typical Transwell images of chemotaxis of peripheral monocytes, isolated from patients with TBI/non-TBI, toward TBI/non-TBI CSF, or SH-SY5Y cell line conditioned medium (CM). **B** The chemotaxis number of peripheral monocytes, isolated from patients with TBI/non-TBI, toward TBI/non-TBI CSF, or SH-SY5Y cell line CM after 16 h. **P < 0.01, determined by paired *t* tests. **C** Levels of CXCL1, CXCL2, and CXCL8 in CSF of TBI or non-TBI patients. The dashed line represents average value of each chemokine in non-TBI CSF. *P < 0.05, **P < 0.01, TBI vs. non-TBI, determined by unpaired t tests or Mann–Whitney tests. **D** Typical Transwell images of chemotaxis of peripheral monocytes, isolated from patients with TBI/non-TBI and pretreated by DMSO (vehicle control) or SB225002, toward TBI CSF. **E** The chemotaxis number of peripheral monocytes, isolated from patients with TBI/non-TBI and pretreated by DMSO (vehicle control) or SB225002, toward TBI CSF after 16 h. **P < 0.01, determined by paired t tests. Data are presented as mean with SEM

We further explored the role of CXCR2 in monocyte chemotaxis in the CSF. We first isolated peripheral monocytes from TBI and non-TBI patients (hereinafter referred to as TBI/non-TBI monocytes) and detected their chemotactic activity in the CSF of TBI/non-TBI patients. The effect of TBI CSF on the recruitment of TBI/non-TBI monocytes was higher than that of non-TBI CSF (Fig. 4A, B). Compared with non-TBI monocytes, monocytes from patients with TBI had higher chemotactic activity in the CSF of both TBI and non-TBI patients (Fig. 4A, B). Moreover, compared with non-TBI monocytes, monocytes from TBI patients showed higher chemotactic activity in the conditioned medium of the SH-SY5Y neuroblastoma cell line (Fig. 4A, B).

To explore whether CXCR2 drives monocyte chemotaxis to the CSF, we first analyzed CXCR2 ligands, including CXCL1, CXCL2, and CXCL8, in the CSF of TBI patients. All these chemokines were detected in TBI CSF after surgery, and overexpressed compared with the average value of those in non-TBI CSF (Fig. 4C). We then pretreated monocytes with the CXCR2 antagonist SB225002 before the chemotaxis assay, and found that the chemotaxis of both TBI/non-TBI monocytes toward TBI CSF was weakened when CXCR2 signaling was inhibited (Fig. 4D, E).

### CXCR2 promotes monocyte-mediated ICD of neural cells

**Fig. 5:**
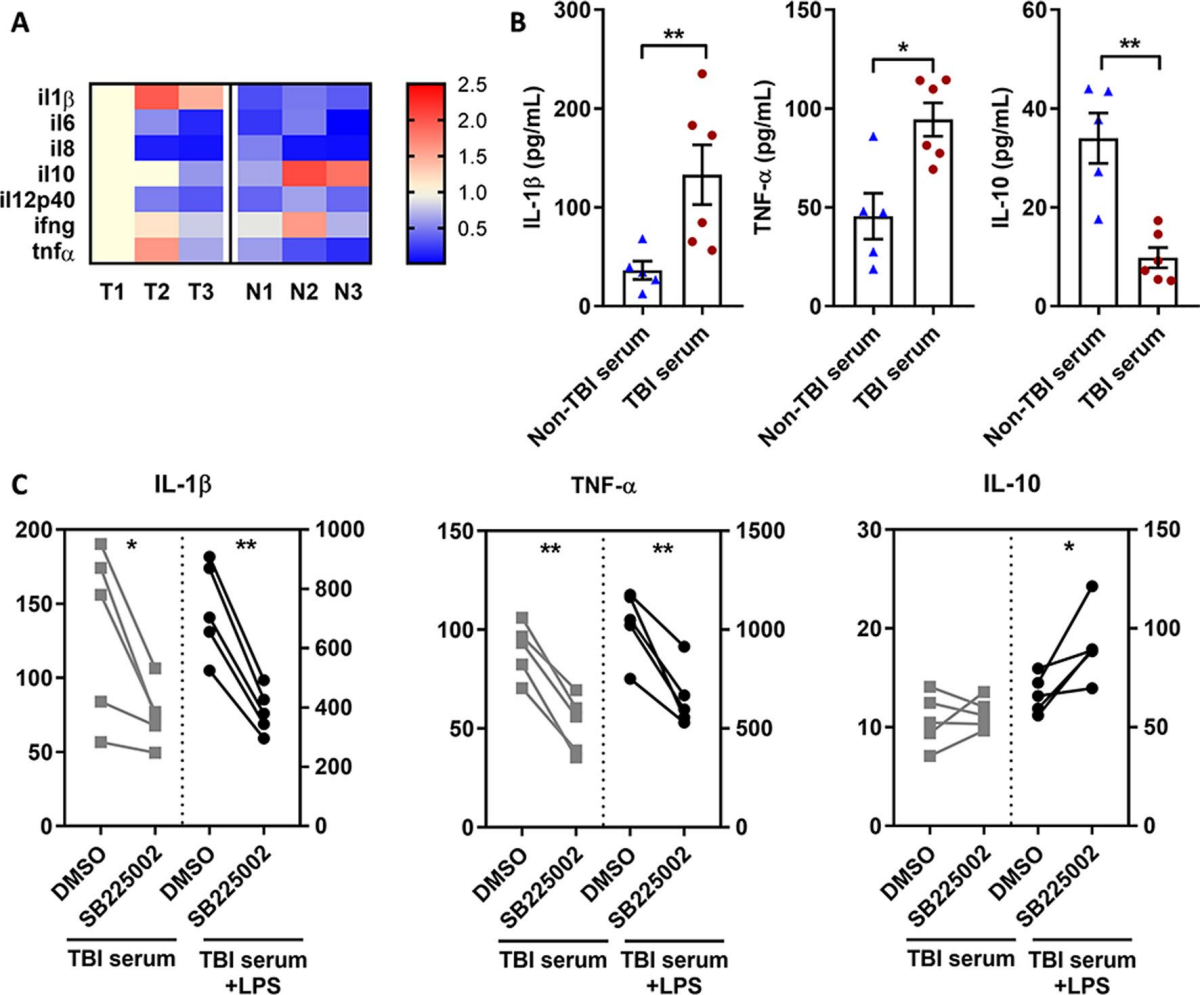
CXCR2 promotes inflammatory cytokine secretion of monocytes and enhanced monocyte-mediated immunogenic cell death (ICD) of neural cells.** A** mRNA levels of cytokines in peripheral CD14 + monocytes isolated from TBI (T1–T3) or non-TBI (N1–N3) patients. The mRNA level of each cytokine in monocytes of T1 is set as 1.0, and the levels in other patients are presented as the fold change of T1. Red indicates expression higher than T1 and blue indicates expression lower than T1. **B** Levels of IL-1β, TNFα, and IL-10 secreted by peripheral monocytes isolated from healthy volunteers and educated by serum of TBI/non-TBI patients. *P < 0.05, **P < 0.01, determined by Mann–Whitney tests. **(C)** Levels of IL-1β, TNFα, and IL-10 secreted by peripheral monocytes isolated from healthy volunteers, pretreated by DMSO (vehicle control) or SB225002, and educated by serum of TBI/non-TBI patients. The grey dots represent monocytes without lipopolysaccharide (LPS) stimulation and the black dots represent monocytes with LPS stimulation. *P < 0.05, **P < 0.01, determined by paired t tests. Data are presented as mean with SEM

ICD commonly occurs shortly after TBI and is an important mechanism of neuroinflammation. The above results showed that CXCR2 signaling drove the chemotaxis of peripheral monocytes toward the CSF, therefore, we explored whether CXCR2 upregulation in monocytes promoted ICD of neural cells. First, we analyzed the cytokine secretion profile of peripheral monocytes from TBI/non-TBI patients. It was found that the transcription levels of inflammatory cytokines IL-1β and TNF-α in TBI monocytes were significantly higher than those in non-TBI monocytes, whereas the transcription of anti-inflammatory cytokine, IL-10, was lower (Fig. 5A). Higher secretion of IL-1β and TNF-α and lower secretion of IL-10 were also found in TBI serum-educated monocytes than in non-TBI-educated monocytes (Fig. 5B). Pre-treatment with the CXCR2 antagonist SB225002 reversed the promoting effect of TBI serum on the secretion of these inflammatory factors in monocytes (Fig. 5C). Moreover, SB225002 also decreased the secretion of IL-1β and TNF-α, and promoted the secretion of IL-10 in LPS-stimulated, TBI serum-educated monocytes (Fig. 5C).

**Fig. 6:**
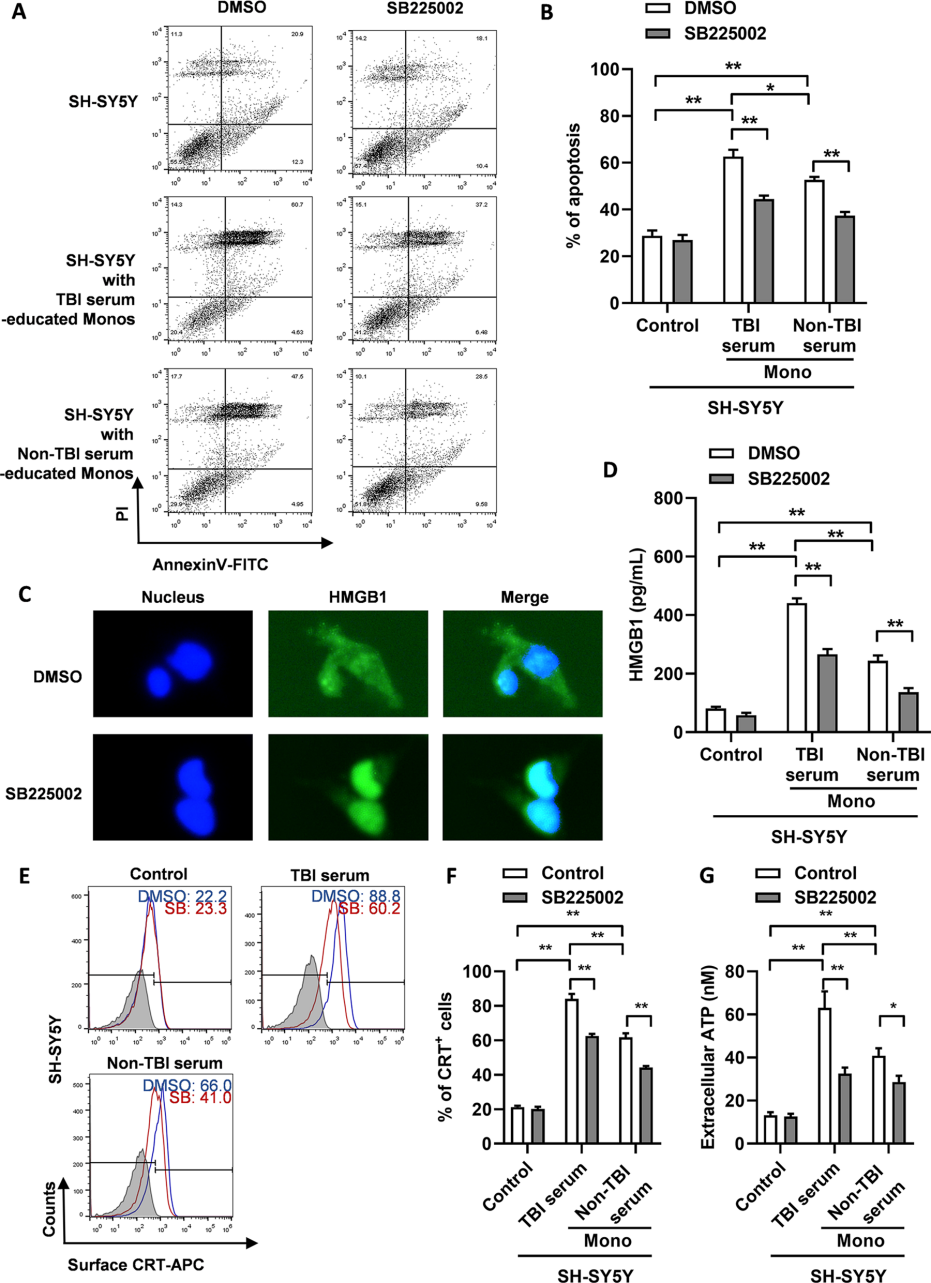
CXCR2 enhances monocyte-mediated ICD of nerve cells.** A** Typical apoptosis images of SH-SY5Y cells co-cultured with TBI/non-TBI serum-educated monocytes, and with DMSO (vehicle control) or SB225002 treatment. **B** Percentage of apoptotic SH-SY5Y cells, co-cultured with TBI/non-TBI serum-educated monocytes and with DMSO (vehicle control) or SB225002 treatment. *P < 0.05, **P < 0.01, determined by paired *t* tests. **C** Typical immunofluorescence images of HMGB-1 expression in SH-SY5Y cells co-cultured with TBI serum-educated monocytes and with DMSO (vehicle control) or SB225002 treatment. **D** HMGB-1 levels in the supernatant of SH-SY5Y cells alone (control), or co-cultured with TBI serum-educated monocytes and with DMSO (vehicle control) or SB225002 treatment. **P < 0.01, determined by paired t tests. **E** Typical flow cytometry results of surface calreticulin (CRT) expression on SH-SY5Y cells alone (control), or co-cultured with TBI/non-TBI serum-educated monocytes and with DMSO (vehicle control) or SB225002 treatment. **F** Percentage of CRT^+^ cells in SH-SY5Y cells alone (control), or co-cultured with TBI/non-TBI serum-educated monocytes and with DMSO (vehicle control) or SB225002 treatment. **P < 0.01, determined by paired *t* tests. **G** Extracellular ATP levels in the supernatant of SH-SY5Y cells alone (control), or co-cultured with TBI/non-TBI serum-educated monocytes and with DMSO (vehicle control) or SB225002 treatment. *P < 0.05, **P < 0.01, determined by paired t tests. Data are presented as mean with SEM

As IL-1β and TNF-α are closely associated with the ICD process, we further analyzed whether TBI monocytes promoted ICD of neural cells. We found that, when co-cultured with TBI serum-educated monocytes, the proportion of apoptotic SH-SY5Y cells induced by H_2_O_2_ was higher than that co-cultured with non-TBI serum-educated monocytes (Fig. 6A, B). SB225002 diminished the proportion of apoptotic SH-SY5Y cells co-cultured with both TBI/non-TBI serum-educated monocytes (Fig. 6A, B). Immunofluorescence staining showed that in SH-SY5Y cells co-cultured with TBI serum-educated monocytes, high mobility group box 1 (HMGB1) was distributed from the nucleus to the cytoplasm, whereas when SB225002 was added, HMGB1 expression was mainly concentrated in the nucleus (Fig. 6C). Higher HMGB1 levels were also found in the supernatant of SH-SY5Y cells co-cultured with TBI serum-educated monocytes, and supplementation with SB225002 decreased HMGB1 levels in the co-culture system containing either TBI/non-TBI serum-educated monocytes (Fig. 6D). SB225002 also decreased calreticulin (CRT) surface expression (Fig. 6E, F) and decreased ATP secretion (Fig. 6G) of SH-SY5Y cells co-cultured with TBI/non-TBI serum-educated monocytes. The above experiments suggest that TBI-educated monocytes promote ICD of neural cells, and CXCR2 signaling promotes this process.

## Discussion

Inflammation and cell death are the core mechanisms of secondary injury in TBI. Monocyte-macrophages are promising targets for TBI because of their important roles in neuroinflammation. However, the regulation of monocyte movement and function in TBI has not been fully elucidated. In this study, we found that CXCR2 expression on classical subsets of monocytes in the peripheral blood and CSF of TBI patients was transiently upregulated after surgery, which was related to the poor short-term prognosis of patients. In vitro experiments showed that the serum and CSF of TBI patients promoted CXCR2 expression in peripheral monocytes, and the anti-inflammatory drug dexamethasone reversed this promoting effect. CXCR2 mediates the migration of peripheral monocytes toward the CSF of TBI patients. The expression of IL-1β and TNF-α in peripheral monocytes treated with TBI serum was significantly higher than in those treated with non-TBI serum. The CXCR2 antagonist SB225002 inhibited the expression of pro-inflammatory cytokines. In the indirect co-culture experiment, TBI serum-educated monocytes exacerbated ICD of a neuroblastoma cell line, SH-SY5Y, induced by oxidative stress, and this process could be inhibited by SB225002. Our study is the first to dynamically observe CXCR2 expression in monocytes of TBI patients and to explore the important role of CXCR2 in monocyte-mediated neuronal ICD.

Neutrophils are the first immune cell type targeted for therapeutic intervention in TBI because of their early infiltration in the CNS after TBI and their contribution to tissue damage through oxidative stress [12]. CXCR2, as a major chemokine receptor mediating neutrophil migration, has long been regarded as a potential target. After TBI, the expression of various CXCR2 ligands such as CXCL1, CXCL2, and CXCL8 is upregulated and correlates with a poorer outcome [13]. CXCR2 knockout mice showed reduced neutrophil infiltration and milder injury [14]. However, the expression pattern of CXCR2 after TBI has not been systematically investigated; therefore, the specific role of CXCR2 in the pathological process of TBI has not been clarified. Our study, for the first time, reported CXCR2 overexpression on monocytes of peripheral blood/CSF after TBI. Of note, PBMCs, which contains lymphocytes and monocytes but no neutrophils, were isolated from peripheral blood and analyzed for CXCR2 expression to exclude the interference of neutrophil-expressed CXCR2 on the results. The dynamic changes in CXCR2 expression were also recorded. This provides supplementary evidence for the role of CXCR2 in TBI treatment, and also provides an important reference for the timing of intervention. According to the results, compared with that before surgery, CXCR2 expression in monocytes was upregulated and peaked 1 day after surgery, then decreased gradually, and relatively stabilized 7 days after surgery. Invasive surgery may contribute to CXCR2 overexpression, however, it is more likely to be caused by the pathological mechanism of TBI itself, because CXCR2 expression in TBI patients was significantly higher 1–4 days after surgery than that in patients who underwent cranioplasty in the same period post-surgery. Another important evidence was that no significant differences were observed in post-operative CXCR2 expression in TBI patients who underwent different operation styles. Peripheral immune cells, including monocytes, enter the brain within 1–2 days after TBI [15]. According to the above observations, intervention targeting CXCR2 should be performed immediately after TBI to reduce the infiltration of peripheral monocytes in the CNS and reduce neuroinflammation.

The dynamic expression pattern of CXCR2 observed in this study is consistent with the previously reported changes in various inflammatory cytokines/chemokines in the serum/CSF of TBI patients. Numerous types of these factors, including IL-1β, IL-6, IL-8, MIP-1α, MIP-1β, and IP10, reach their peak within 1–2 days after TBI [16]. The systemic inflammatory status of TBI patients may affect the phenotypes and functions of peripheral monocytes. In the present study, we found that, compared with serum of non-TBI patients, TBI serum significantly promoted CXCR2 expression in peripheral monocytes. Dexamethasone, a commonly used anti-inflammatory drug and NF-κB pathway inhibitor, can reverse the effect of TBI serum on CXCR2 expression in monocytes. Unfortunately, although our results demonstrate that NF-κB signaling may play an important role in CXCR2 overexpression, we have not confirmed the specific factors in the TBI serum/CSF that can activate this pathway in monocytes. We speculate that there are numerous factors that play redundant and complex interactive roles, and neutralization or supplementation of a single factor in vitro cannot completely simulate the effect of TBI serum/CSF. Nevertheless, our results suggest that the systemic inflammatory response in TBI patients is an important factor causing the upregulation of CXCR2 expression in monocytes.

Furthermore, we found that the CXCR2 pathway is related to the overexpression of IL-1β and TNFα in TBI monocytes. Both IL-1β and TNFα are recognized inducers of ICD [17, 18]. In vitro, TBI-educated monocytes promoted the apoptosis of SH-SY5Y cells induced by oxidative stress, compared with non-TBI-educated ones, and the CXCR2 antagonist SB225002 reversed the promoting effect of TBI-educated monocytes. Of note, SB225002 decreased HMGB1 secretion, CRT surface exposure and ATP release, which are ICD-associated damage-associated molecular patterns, in SH-SY5Y cells induced by TBI-educated monocytes. Overexpression of extracellular HMGB1 was found in plasma, serum and CSF of TBI patients from several distinct clinical studies [19–21]. Extracellular HMGB1 interacts with the toll-like receptor 4 (TLR4) and the receptor for advanced glycation end products (RAGE), namely expressed by microglia, resulting in immunocytes migration and excessive release of pro-inflammatory cytokines [22]. Extracellular HMGB1 is a plausible biomarker to predict neuroinflammation-associated secondary injury and long-term outcomes after TBI [23]. Targeting HMGB1 has been reported to improve short-term and chronic outcomes in multiple preclinical TBI models [23]. The above studies supported the role of CXCR2 antagonist as promising treatment against TBI by inhibiting monocyte-mediated ICD of nerve cells. Notably, SB225002 significantly inhibited the effect of LPS on the overexpression of IL-1β and TNFα in TBI-educated monocytes. Previous studies have found that LPS stimulation promotes the inflammatory response of microglia in TBI mice and increases the risk of depression-like behavior and cognitive impairment [24, 25], and therefore drove the important effects of subsequent systemic immune activation, induced by post-injury infection, on the long-term outcome of patients. Thus, inhibition of CXCR2 signaling may improve the long-term outcome of TBI patients, as it inhibits the responsiveness of peripheral monocytes to LPS. Unfortunately, due to the difficulty of follow-up, we have not received sufficient long-term outcomes of TBI patients involved in our study, and we will further analyze this relationship in future studies.

## Conclusion

Our results show that CXCR2 overexpression in the classical subset of peripheral monocytes soon after TBI is involved in monocyte recruitment to the CNS and monocyte-mediated neuronal ICD, which is related to the short-term prognosis of TBI patients. This study suggests that the CXCR2 pathway may be an important mechanism of monocyte-mediated neuroinflammation and may become an important target to reduce secondary injury after TBI.

## Supplementary Information

**Additional file 1: Table S1.** Demographic and clinical data of TBI patients.

**Additional file 2: Fig. S1. **CXCR2 expression in peripheral monocytes of TBI patients with different intervals from TBI to surgery. **A** Preoperative ΔMFI value of CXCR2 in peripheral CD14^+^ monocytes of TBI patients with different intervals from TBI to surgery (≤ 12 h, 13-24 h and > 24 h). **B** Postoperative ΔMFI value of CXCR2 in TBI patients with different intervals from TBI to surgery. **C** Average values of pre- or post-operative (within 3 days or 4–5 days) CXCR2-positive percentages and ΔMFI values in TBI patients with different intervals from TBI to surgery. *P < 0.05, 13-24 h group vs. > 24 group, determined by Mann–Whitney tests.

**Additional file 3: Fig. S2. **CXCR2 expression in peripheral monocytes of TBI patients with different operation style. **A** Preoperative ΔMFI value of CXCR2 in peripheral CD14^+^ monocytes of TBI patients with different operation style (aspiration, craniotomy and decompressive craniectomy). **B** Postoperative ΔMFI value of CXCR2 in TBI patients with different operation style. **C** Average values of pre- or post-operative (within 3 days or 4–5 days) CXCR2-positive percentages and ΔMFI values in TBI patients with different operation style. DC, decompressive craniectomy.

## Data Availability

The data sets used in this study are available from the corresponding author upon reasonable request.

## Abbreviations

BBB: Blood–brain barrier
CRT: Calreticulin
CNS: Central nervous system
CSF: Cerebrospinal fluid
ELISA: Enzymes linked immunosorbent assay
GCS: Glasgow Coma Score
GOS: Glasgow Outcome Score
HMGB1: High mobility group box 1
ICD: Immunogenic cell death
ICP: Intracranial pressure
LPS: Lipopolysaccharide
mAb: Monoclonal antibodies
MFI: Mean fluorescence intensity
PBMCs: Peripheral blood mononuclear cells
TBI: Traumatic brain injury
