# Supplementary material for: Transient post-operative overexpression of CXCR2 on monocytes of traumatic brain injury patients drives monocyte chemotaxis toward cerebrospinal fluid and enhances monocyte-mediated immunogenic cell death of neurons in vitro
Source: J Neuroinflammation. 2022 Jun 29;19:171. doi: 10.1186/s12974-022-02535-6 (PMC9245242; doi:10.1186/s12974-022-02535-6)
Supplement: Supplementary file 1 — Additional file 1: Table S1. Demographic and clinical data of TBI patients. [file 12974_2022_2535_MOESM1_ESM.docx]

**Supplemental Table 1. Demographic and clinical data of TBI patients**

| **Characteristics** | **mean±SD or n (%)** |
| --- | --- |
| **Gender** |  |
| Male | 61 (82.43) |
| Female | 13 (17.57) |
| **Age(years)** |  |
|  | 50.91±15.33 |
| **Hospital day (days)** |  |
|  | 15.40±7.52 |
| **Injury mechanism** |  |
| Fall | 38 (51.35) |
| Traffic accident | 29 (39.19) |
| Others | 7 (9.46) |
| **Dilated pupil** |  |
| Diameter of both pupils <4 mm | 58 (78.38) |
| Diameter of 1 pupil ≥4 mm | 6 (8.11) |
| Diameter of both pupils ≥4 mm | 10 (13.51) |
| **Pupillary reactivity** |  |
| Brisk | 34 (45.95) |
| Sluggish | 22 (29.73) |
| Nonreactive | 18 (24.32) |
| **GCS score (Admission)** |  |
| 3~8 | 42 (56.76) |
| 9~12 | 16 (21.62) |
| 13~15 | 16 (21.62) |
| **Injury style** |  |
| Unilateral | 58 (78.38) |
| Bilateral | 16 (21.62) |
| **Interval from injury to surgery (h)** |  |
|  | 21.79±19.89 |
| **Operation style** |  |
| Aspiration | 27 (36.49) |
| Craniectomy | 22 (29.73) |
| Decompressive craniectomy | 25 (33.78) |
| **Tracheotomy** |  |
| Yes | 18 (24.32) |
| No | 56 (75.68) |
