# Supplementary figures and images for: Transient post-operative overexpression of CXCR2 on monocytes of traumatic brain injury patients drives monocyte chemotaxis toward cerebrospinal fluid and enhances monocyte-mediated immunogenic cell death of neurons in vitro
Source: J Neuroinflammation. 2022 Jun 29;19:171. doi: 10.1186/s12974-022-02535-6 (PMC9245242; doi:10.1186/s12974-022-02535-6)

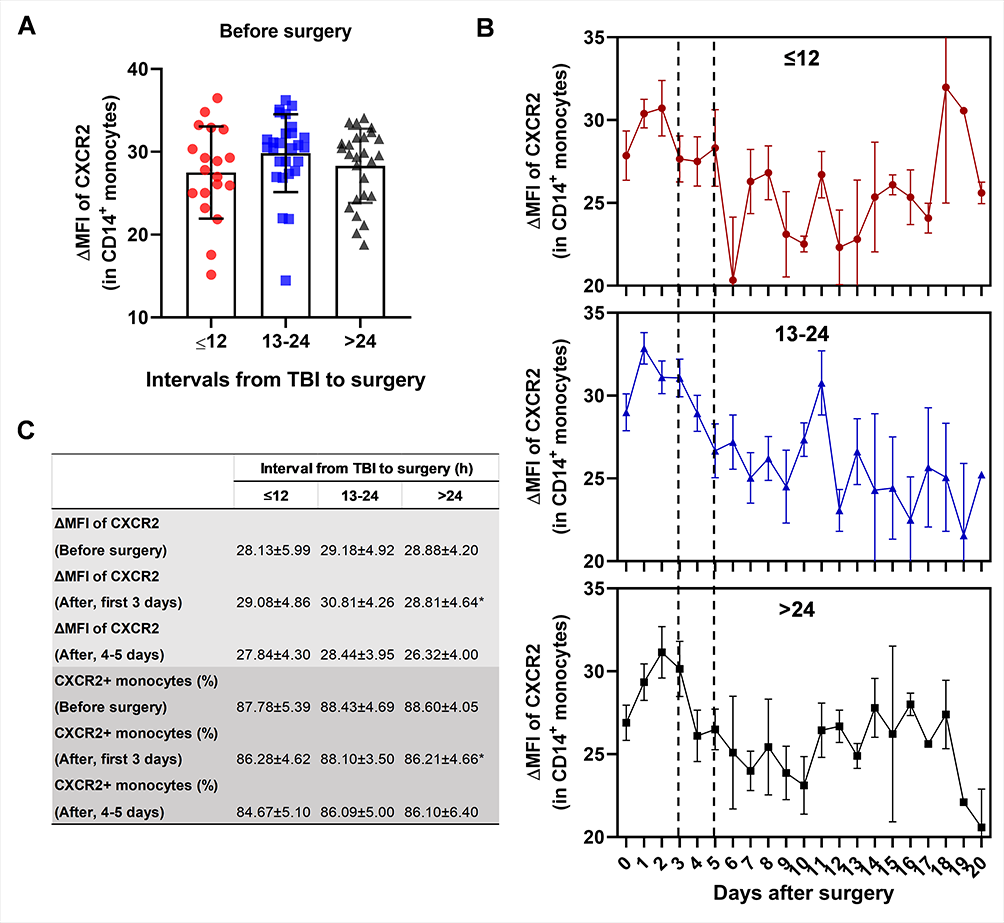

Supplement: Supplementary file 2 — Additional file 2: Fig. S1. CXCR2 expression in peripheral monocytes of TBI patients with different intervals from TBI to surgery. A Preoperative ΔMFI value of CXCR2 in peripheral CD14+ monocytes of TBI patients with different intervals from TBI to surgery (≤ 12 h, 13-24 h and > 24 h). B Postoperative ΔMFI value of CXCR2 in TBI patients with different intervals from TBI to surgery. C Average values of pre- or post-operative (within 3 days or 4–5 days) CXCR2-positive percentages and ΔMFI values in TBI patients with different intervals from TBI to surgery. *P < 0.05, 13-24 h group vs. > 24 group, determined by Mann–Whitney tests. [file 12974_2022_2535_MOESM2_ESM.tif]

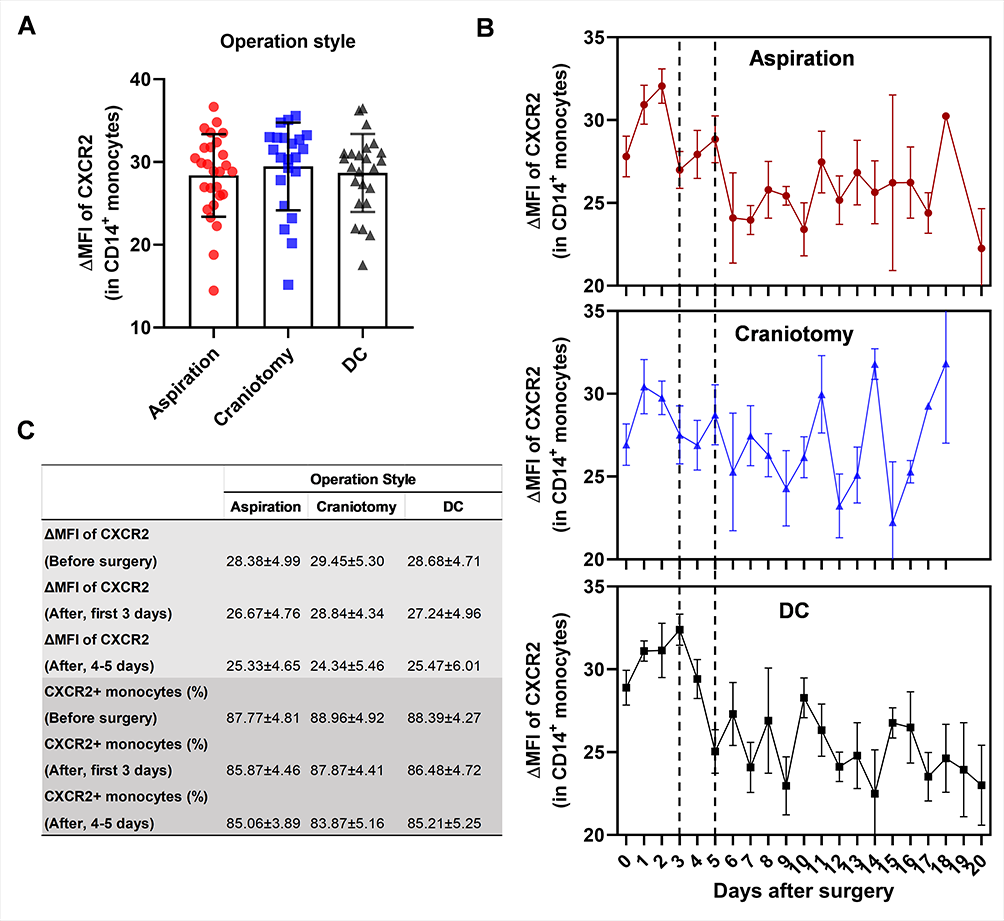

Supplement: Supplementary file 3 — Additional file 3: Fig. S2. CXCR2 expression in peripheral monocytes of TBI patients with different operation style. A Preoperative ΔMFI value of CXCR2 in peripheral CD14+ monocytes of TBI patients with different operation style (aspiration, craniotomy and decompressive craniectomy). B Postoperative ΔMFI value of CXCR2 in TBI patients with different operation style. C Average values of pre- or post-operative (within 3 days or 4–5 days) CXCR2-positive percentages and ΔMFI values in TBI patients with different operation style. DC, decompressive craniectomy. [file 12974_2022_2535_MOESM3_ESM.tif]
